# Immune Imprinting, Non-Durable Hybrid Immunity, and Hybrid Immune Damping Following SARS-CoV-2 Primary Vaccination with BNT162b2 and Boosting with mRNA-1273

**DOI:** 10.3390/vaccines13030310

**Published:** 2025-03-13

**Authors:** Alejo Erice, Néstor Nuño, Lola Prieto, Cristina Caballero

**Affiliations:** 1 Department of Internal Medicine, Hospital Asepeyo, 28823 Coslada, Spain; 2 Facultad de Medicina, Universidad Francisco de Vitoria, 28223 Pozuelo de Alarcón, Spain; 3 Independent Researcher, 28400 Madrid, Spain; nuno7@hotmail.com; 4 Unidad de Apoyo a la Investigación, Facultad de Medicina, Universidad Francisco de Vitoria, 28223 Pozuelo de Alarcón, Spain; lola.prieto@ufv.es; 5 Clinical Diagnostic Laboratory, Hospital Asepeyo, 28823 Coslada, Spain; ccaballero1983@hotmail.com

**Keywords:** SARS-CoV-2, SARS-CoV-2 vaccine, BNT162b2, mRNA-1273, SARS-CoV-2 antibody humoral immune response, SARS-CoV-2 T-cell response, immune imprinting, immune damping

## Abstract

**Background/Objectives:** Long-term studies on the immune response following multiple doses of SARS-CoV-2 mRNA vaccines remain limited. **Methods:** Secondary analyses of data from a cohort of non-immunocompromised subjects who received two doses of BNT162b2 (primary vaccination) and a booster with mRNA-1273 nine months later. Antibodies targeting the receptor-binding domain of the S1 subunit of the SARS-CoV-2 spike (anti-RBD) were measured at eight time points during follow-up; the SARS-CoV-2-specific T cell response was measured 16 and 25 months after primary vaccination using an interferon-γ release assay. **Results:** During the 9-month follow up period after primary vaccination and before the mRNA-1273 booster, anti-RBD were significantly higher at all time points in subjects with documented SARS-CoV-2 infection before the first study time point (previously infected subjects; n = 50) compared to naïve subjects (n = 208; *p* < 0.05). During a 16-month follow up period following the mRNA-1273 booster, anti-RBD were lower at all time points in previously infected subjects (n = 21) compared to naïve subjects (n = 109), although the differences were non-significant. Breakthrough SARS-CoV-2 infections increased over time in both groups, particularly after the mRNA-1273 booster. Most participants had a persistent SARS-CoV-2 specific T cell response regardless of prior infection. **Conclusions:** These findings suggest a modulating effect of previous SARS-CoV-2 infection on the humoral immune response to mRNA vaccination, a non-durable hybrid immunity following mRNA vaccination in previously infected subjects, and attenuation of the humoral immune response (immune damping) after repeated exposure to SARS-CoV-2 antigens through mRNA vaccination and/or infection.

## 1. Introduction

Severe acute respiratory syndrome coronavirus 2 (SARS-CoV-2) messenger RNA (mRNA) vaccines are effective at protecting against severe disease, hospitalization, and death caused by SARS-CoV-2 infection [1,2]. However, the efficacy of mRNA vaccines decreases over time due to waning immunity and the emergence of highly transmissible SARS-CoV-2 variants capable of evading vaccine induced immunity, resulting in breakthrough infections [3,4,5]. To extend vaccine induced immune protection and overcome the immune escape of emerging variants of the virus, additional doses of mRNA vaccines given as boosters and mRNA vaccines designed to target new SARS-CoV-2 variants have been recommended [6,7,8]. While additional doses of mRNA vaccines have significantly reduced COVID-19-related hospitalizations and deaths, less severe SARS-CoV-2 breakthrough infections continue occurring among vaccinated subjects [9].

Long-term studies on the humoral immune response following multiple doses of SARS-CoV-2 vaccines remain limited. Following primary vaccination with mRNA vaccines, there is a strong neutralizing antibody response against SARS-CoV-2 that wanes by three to six months and declines further by eight months [10]. While neutralization assays are the gold standard for assessing this response, they are time consuming and yield heterogeneous results that are difficult to interpret. Antibodies targeting the receptor-binding domain of the S1 subunit of the spike protein of SARS-CoV-2 (anti-RBD antibodies) are generated following infection and mRNA vaccination; these antibodies can be readily measured using automated commercially available laboratory methods. Strong correlations between anti-RBD antibody levels and viral neutralizing activity have been well established, supporting the use of anti-RBD antibodies as a reliable surrogate marker for neutralizing activity and protection against SARS-CoV-2 infection [11,12,13,14].

In a previous study on the immune response following SARS-CoV-2 mRNA vaccines, we analyzed the dynamics of the anti-RBD antibody response over a two-year period in a cohort of non-immunocompromised subjects who received two doses (primary vaccination) of the mRNA vaccine BNT162b2 (Comirnaty, BioNTech Manufacturing GmbH, Mainz, Germany), followed by a booster dose of mRNA-1273 (Spikevax, ModernaTx, Inc., Norwood, MA, USA), and a dose of the Pfizer-BioNTech bivalent Omicron-adapted BNT162b2 mRNA vaccine (Mainz, Germany) [15]. Overall, anti-RBD antibody titers declined during the initial nine months of follow-up, increased after the mRNA-1273 booster, and declined gradually thereafter. In addition, the incidence of breakthrough SARS-CoV-2 infections increased over time. We hypothesized that the kinetics of the anti-RBD antibody response and the occurrence of SARS-CoV-2 breakthrough infections during follow up may have reflected immune modulation driven by a prior SARS-CoV-2 infection and repeated exposures to viral antigens through mRNA vaccines and breakthrough infections. To further explore those hypotheses, we conducted secondary analyses of data collected from this cohort of healthy non-immunocompromised adults.

We found that SARS-CoV-2 infection occurring before or shortly after primary vaccination with BNT162b2 modulated the vaccine-elicited anti-RBD antibody response. Specifically, previously infected subjects had higher anti-RBD antibody titers after primary mRNA vaccination compared to naïve subjects; however, their titers declined to lower levels during long-term follow up after the mRNA-1273 booster, suggesting a non-durable humoral hybrid immune response against the virus. We also observed that breakthrough SARS-CoV-2 infections increased over time, particularly after the mRNA-1273 booster. Most participants had a durable SARS-CoV-2 specific T cell response regardless of prior infection. In addition to immune imprinting, our results imply that hybrid immune damping may have resulted from repeated exposures to SARS-CoV-2 antigens through additional mRNA vaccinations and breakthrough infections.

## 2. Materials and Methods

### 2.1. Study Design

Secondary analyses of data on the immune response following the administration of SARS-CoV-2 mRNA vaccines in a cohort of non-immunocompromised subjects who received two doses of BNT162b2 (primary vaccination) followed by a booster with mRNA-1273 [15]. Two subsets were analyzed: (1) data obtained during the nine-month period elapsed from primary vaccination with BNT162b2 to the administration of the mRNA-1273 booster and (2) data obtained during a 16-month period after the mRNA-1273 booster.

### 2.2. Anti SARS-CoV-2 Antibodies

Anti-RBD antibodies were measured using the Architect SARS-CoV-2 IgG II Quant assay (Abbott, Chicago, IL, USA) [15]. Anti-nucleocapsid antibodies (anti-N antibodies) were measured using the Architect SARS-CoV-2 IgG assay (Abbott, Chicago, IL, USA) [15].

### 2.3. SARS-CoV-2 Specific T Cell Response

The SARS-CoV-2-specific T cell response was analyzed using an interferon gamma (IFN-γ) release assay (Quant-T cell SARS-CoV-2 + Quant-T cell ELISA EUROIMMUN, Lübeck, Germany) [15]. Results > 200 milli-international units (mIU)/mL were considered positive. The number of T-lymphocytes in each sample was not determined; therefore, the T cell response could not be quantified based on IFN-γ levels.

### 2.4. SARS-CoV-2 Infections

At each study time point, information was collected on the occurrence of symptomatic SARS-CoV-2 infection (defined as symptoms plus a positive polymerase chain reaction or antigen test in upper respiratory tract samples) since the previous visit. Asymptomatic SARS-CoV-2 infections were diagnosed by the de novo appearance of anti-N antibodies and/or a >5.1% increase in ant-RBD antibodies not justified by vaccination within the previous three months [15].

### 2.5. Statistical Analysis

Changes in anti-RBD antibody titers based on prior infection with SARS-CoV-2 were analyzed using a linear mixed model with a random intercept, adjusted for age, sex, and SARS-CoV-2 breakthrough infections. Anti-RBD antibody titers were presented as geometric mean titer (GMT) ratios and 95% confidence intervals (CI). The Bonferroni correction method (pairwise comparisons) was used to compare anti-RBD antibody titers between groups. Statistical analyses were performed using R 3.6 (R Project for statistical computing) and STATA 15.0 (Stata Corporation, College Station, TX, USA); *p*-values < 0.05 were considered significant.

## 3. Results

### 3.1. Study Population

The first group included 258 subjects [mean age: 45.9 years (SD 11.5); 161 (62%) females and 97 (38%) males] followed for nine months after primary vaccination with BNT-162b2; none received additional mRNA vaccines during that follow up period. The second group included 130 subjects from the first group [mean age: 45.4 years (SD 11.3); 84 (65%) females and 46 (35%) males] followed for 16 months after receiving the mRNA-1273 booster a mean of 296 days (SD 13.7) after primary vaccination with BNT162b2. Blood samples from the first group were collected 1.5, 3, 7 and 9 months after primary vaccination. Blood samples from the second group were collected 4, 7, 10, and 16 months after the mRNA 1273 booster (corresponding to 13, 16, 19, and 25 months from primary vaccination, respectively).

Previously infected subjects were those with documented SARS-CoV-2 infections that occurred before the first study follow up point (1.5 months after primary vaccination with BNT162b2). Naïve subjects were those without evidence of prior SARS-CoV-2 infection at the first study time point.

### 3.2. Kinetics of SARS-CoV-2 Anti-RBD Antibodies

**Figure 1:**
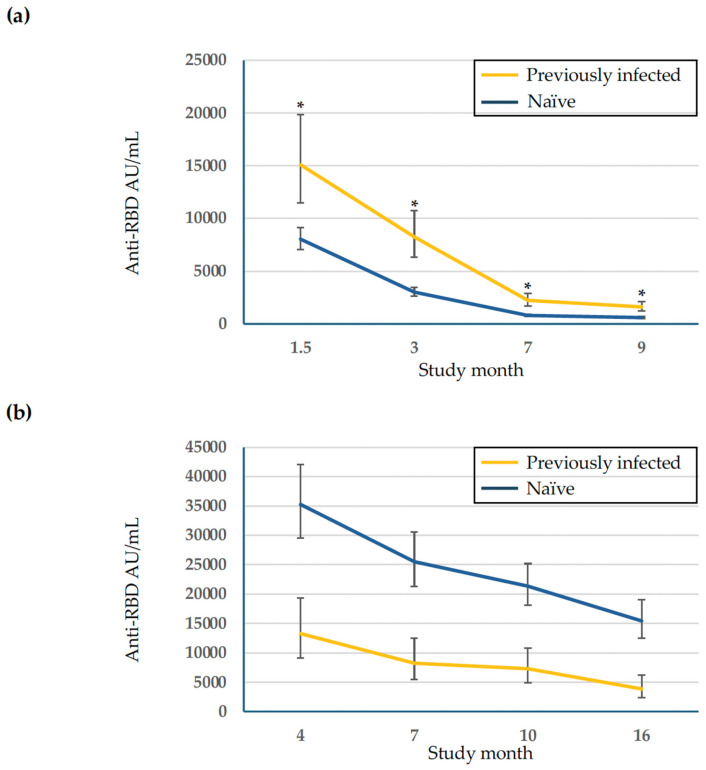
Evolution of anti-RBD antibodies. (**a**) Anti-RBD antibody titers [GMT (95% CI)] following primary vaccination with BNT162b2; (**b**) Anti-RBD antibody titers [GMT (95% CI)] following the mRNA-1273 booster. Vertical lines represent upper and lower 95% CI limits at each time point. Previously infected subjects had documented SARS-CoV-2 infection that occurred before the first study time point (1.5 months after primary BNT162b2 vaccination. Naïve subjects had no evidence of prior SARS-CoV-2 infection at the first study time point. * Significantly higher anti-RBD antibody titers at indicated time points (*p* < 0.05).

The evolution of anti-RBD antibody titers is shown in Table 1 and Table 2, and Figure 1. During the 9-month follow up period after primary vaccination with BNT162b2, anti-RBD antibodies declined by 89% in previously infected subjects and by 93% in naïve subjects. Anti-RBD antibody titers were significantly higher at all time points during this period in previously infected subjects compared to anti-RBD titers in naïve subjects (*p* < 0.05) (Table 1).

During the 16-month follow up period following the mRNA-1273 booster, anti-RBD antibodies declined by 50% in previously infected subjects and by 23% in naïve subjects. Anti-RBD antibodies were lower at all time points in previously infected subjects compared to anti-RBD antibody titers in naïve subjects, although the differences were not significant (Table 2).

### 3.3. SARS-CoV-2 Breakthrough Infections During Follow-Up

SARS-CoV-2 breakthrough infections during follow up are shown in Table 3. Thirty-six (18%) breakthrough infections occurred during the 9-month period following primary vaccination with BNT162b2; 8 (22%) were symptomatic and 28 (78%) were asymptomatic. Overall, 160 (82%) breakthrough infections occurred during the 16-month follow up period after the mRNA-1273 booster; 74 (46%) were symptomatic and 86 (54%) were asymptomatic. All symptomatic infections in both groups were mild.

During the 9-month period after primary vaccination with BNT162b2, rates of SARS-CoV-2 breakthrough infection were higher at all time points among previously infected subjects compared to infection rates in naïve subjects, except at the 3-month time point (Table 3). The dominant circulating SARS-CoV-2 variants during this period were Alpha (B.1.1.7) from primary vaccination with BNT162b2 throughout the 1.5-month time point and Delta (B.1.617.2) from 1.5 months through 9 months following primary vaccination with BNT162b2 [16].

During the 16-month period following the mRNA-1273 booster, rates of SARS-CoV-2 breakthrough infection were higher at the 4-month and 7-month time points among subjects previously infected with SARS-CoV-2 compared naïve subjects, whereas infection rates were higher in naïve subjects at the 10-month and 16-month time points (Table 3). Omicron (B.1.1.529) and its subvariants were the dominant circulating SARS-CoV-2 variants during this 16-month period [16].

### 3.4. SARS-CoV-2-Specific T Cell Response

The SARS-CoV-2-specific T cell response was analyzed in blood samples collected 16 months and 25 months after primary vaccination with BNT162b2 (Table 4).

The SARS-CoV-2-specific T cell response was measured at both time points in 69 subjects: 58 (84%) had a positive result both times, 6 (9%) had an initial positive result followed by a negative result, and 5 (7%) had an initial negative result followed by a positive result. Overall, the frequencies of positive and negative SARS-CoV-2-specific T cell responses between the two measurements were not statistically significant.

## 4. Discussion

In this study, we analyzed the evolution of anti-RBD antibodies and SARS-CoV-2 breakthrough infections in healthy adults over a 9-month period following primary vaccination with BNT162b2 and over a 16-month period after the same individuals received a booster with the mRNA-1273 vaccine. We found that anti-RBD antibodies declined over time following primary vaccination with BNT162b2 and the mRNA 1273 booster. We also observed that prior SARS-CoV-2 infection was associated with significantly higher titers of anti-RBD antibodies at all time points during the 9-month period following primary vaccination; however, this trend reversed during the 16-month follow up period after the mRNA-1273 booster, with lower anti-RBD antibody titers at all time points in previously infected subjects compared to naïve individuals. We also found that breakthrough SARS-CoV-2 infections increased over time, particularly after the mRNA booster. Finally, a durable SARS-CoV-2 specific T cell response was found in most of the study participants, irrespective of prior infection.

Studies on the long-term evolution of anti-RBD antibodies in healthy individuals following repeated doses of SARS-CoV-2 mRNA vaccines are limited. The overall decline of anti-RBD antibodies over time following mRNA vaccination observed in our study is consistent with the findings from others [14,17]. In a study of 1024 healthy individuals who received SARS-CoV-2 mRNA vaccines, anti-RBD antibodies progressively declined over a 16-month period following vaccination [14]. In a large observational longitudinal study of 501 healthy subjects followed over three years, primary vaccination with a SARS-CoV-2 mRNA vaccine induced a durable anti-RBD antibody response characterized by two distinct phases: an initial rapid decay followed by a stabilization phase with a very slow decay in antibody titers [17]. Similarly to our study, anti-RBD antibody titers were significantly higher during the first nine months following vaccination in subjects with prior SARS-CoV-2 infection compared to naïve subjects [17]. A large observational longitudinal study in health care workers followed over 18 months also found that prior SARS-CoV-2 infection was associated with persistently higher anti-RBD antibody titers compared to antibody titers in the absence of prior infection [18]. The higher anti-RBD antibody titers in subjects with previous SARS-CoV-2 infection reflects hybrid immunity, where the combination of natural infection and vaccination results in a stronger immune response than immunity derived from infection alone or vaccine-derived immunity alone [19,20].

Following the mRNA-1273 booster, anti-RBD antibody titers in subjects with SARS-CoV-2 infection before primary vaccination were consistently lower than those in naïve subjects at all time points during follow up. In contrast to other reports [19,20], this finding suggests that the hybrid immune response (conferred by a prior SARS-CoV-2 infection and mRNA vaccination) may not be durable. Other studies have similarly reported a reduced anti-RBD antibody response in vaccinated subjects with a history of SARS-CoV-2 infection before primary vaccination, suggesting a modulating effect of prior infection on the anti-RBD antibody response to additional doses of mRNA vaccines [21,22,23].

SARS-CoV-2 breakthrough infections increased over time in our study. During the 9-month period following primary vaccination with BNT162B2, the dominant circulating SARS-CoV-2 variants were Alpha (from primary vaccination through the 1.5-month time point) and Delta (from 1.5 months to 9 months following primary vaccination). The few breakthrough infections observed during this period were most likely the consequence of waning of vaccine-induced protection and the immune evasiveness of the dominant Delta (B.1.167.2) variant [15,24,25]. Breakthrough SARS-CoV-2 infections increased steadily during the 16-month period following the mRNA-1273 booster, when Omicron (B.1.1.529) was the dominant circulating variant. Breakthrough infections occurred irrespective of prior infection with SARS-CoV-2; although the differences were not significant, rates of breakthrough infections were higher among previously infected individuals compared to naïve individuals. The increasing breakthrough infection rate over time suggests waning immunity in both naïve and previously infected subjects that persisted after the mRNA-1273 booster.

The lower anti-RBD antibody titers and increasing breakthrough infection rates observed among previously infected individuals vaccinated with BNT162b2 and mRNA-1273 may indicate hybrid immune damping. This term refers to the combination of diminished antibody responses and reduced immune protection in recipients of multiple mRNA vaccine doses following Omicron (B.1.1.529) infection [22,23,26]. Hybrid immune damping is considered an unexpected consequence of immune imprinting resulting from infection with an early SARS-CoV-2 variant before mRNA vaccination [22] and could provide a partial explanation for the findings in our study.

A potential mechanism contributing to the increasing rates of SARS-CoV-2 breakthrough infections among mRNA vaccinated individuals during the period of Omicron (B.1.1.529) dominance is antibody-dependent enhancement of infection (ADE). ADE occurs when antibodies facilitate viral entry into host cells rather than neutralizing the virus, typically through interactions between virus–antibody complexes and cellular receptors [27]. Studies on ADE in the context of SARS-CoV-2 infections and mRNA vaccination have shown inconsistent results. In vitro studies have shown ADE activity in sera from a limited number of individuals following mRNA-1273 vaccination [28]. In those experiments, the same antibodies exhibited both neutralization and ADE activities, with ADE observed within relatively narrow antibody concentrations that failed to effectively neutralize Omicron (B.1.1.529) [28,29]. Mild ADE has also been demonstrated when serum samples obtained from individuals at different stages of mRNA vaccination were incubated with Omicron (B.1.1.529); however, ADE was not observed with samples obtained after completion of the full mRNA vaccination scheme [30]. Comprehensive review studies and meta-analyses have not identified ADE as a significant clinical issue in mRNA vaccinated populations [26,31]. Nevertheless, ongoing surveillance of mRNA vaccinated individuals remains essential to identify and mitigate ADE as well as other potential risks.

Additional factors that could contribute to the waning of anti-RBD antibodies after exposures to SARS-CoV-2 antigens through infection and/or mRNA vaccination have been described [32,33]. One study in unvaccinated subjects found that SARS-CoV-2 infection failed to generate spike-specific antibody-secreting long-lived plasma cells (LLPCs) in the bone marrow [32]. In a more recent study, analyses of bone marrow samples from healthy adults 2.5 to 33 months after receiving two to five doses of mRNA vaccines showed that SARS-CoV-2 spike-specific antibody-secreting cells were largely absent from the LLPC population [33]. Since bone marrow LLPCs are responsible for providing a durable antibody response after infection or vaccination, these findings provide an additional mechanistic explanation for the limited duration of the systemic antibody responses observed after SARS-CoV-2 mRNA vaccines.

Long term studies on the SARS-CoV-2 specific T cell response following multiple doses of mRNA vaccines are limited. In addition, studies on the T cell response elicited by the combined effect of vaccination with multiple doses of mRNA vaccines and SARS-CoV-2 infections (hybrid immunity) are also scarce. Current evidence indicates that after two doses of mRNA vaccines, SARS-CoV-2 specific T cell responses are detected in nearly all individuals, with memory T cells playing a critical role in long-term protection against severe disease, even as antibody-mediated neutralization is reduced by waning immunity and/or the emergence of immune-evasive variants of the virus [34,35,36]. In our study, a positive SARS-CoV-2 specific T cell response was observed in most participants 16 months and 25 months after primary vaccination with BNT162b2, suggesting a durable SARS-CoV-2 specific T cell response irrespective of prior infection with SARS-CoV-2. Of note, a SARS-CoV-2 specific T cell response was present in individuals who had been repeatedly exposed to viral antigens through mRNA vaccinations and breakthrough infections; although the sample size was small, our data do not suggest that repeated exposure to SARS-CoV-2 antigens over time caused T cell exhaustion or impaired the cellular immune response [37].

A major limitation of our study is that our observations were based on whole anti-RBD antibody measurements; variant-specific anti-RBD antibodies and neutralization analyses were not performed. However, strong correlations between anti-RBD antibody levels and viral neutralizing activity have been well established, making anti-RBD antibodies a reliable surrogate marker for neutralizing activity and protection against SARS-CoV-2 infection [11,12,13,14]. Additionally, whole anti-RBD antibodies have been shown to correlate with variant-specific anti-RBD antibodies and neutralizing antibodies [21,22,23], with some discordances reported for Delta (B.617.2), P.1 (Gamma), and Omicron (B.1.1.529) [22]. Therefore, despite the absence of variant-specific anti-RBD antibodies and neutralization data in our study, we believe that our observations remain valid.

The additional limitations of our study are the following: (1) only healthy non-immunocompromised middle-aged workers at a single site were included; therefore, the findings might not be extended to other populations; (2) data were not available from all study participants at all time points due to a small drop-out rate; (3) exposure of our participants to SARS-CoV-2 antigens was limited to three doses of mRNA vaccines and the infections that occurred during follow-up; exposure to SARS-CoV-2 antigens in advanced countries is now higher because additional doses of mRNA vaccines are being administered and there are ongoing breakthrough SARS-CoV-2 infections; (4) the number of participants with a history of SARS-CoV-2 infection at baseline was low; (5) baseline anti-RBD antibodies were not measured until 1.5 months after primary vaccination with BNT162b2; (6) information on the early SARS-CoV-2-specific T cell response was not available because measurements were conducted months after primary vaccination and the administration of the mRNA-1273 booster; and (7) the qualitative T cell assay we used did not allow quantification of changes in the magnitude of the SARS-CoV-2 specific T cell response over time.

## 5. Conclusions

Anti-RBD antibodies in healthy adults declined over time following both primary vaccination with BNT162b2 and a booster with mRNA-1273. SARS-CoV-2 infection before or shortly after primary vaccination modulated the anti-RBD antibody response, resulting in significantly higher titers following primary BNT162b2 vaccination that reversed to lower levels during long-term follow-up after the mRNA-1273 booster, suggesting non-durable hybrid immunity. Non-severe breakthrough SARS-CoV-2 infections increased over time and were more frequent following the mRNA-1273 booster. These findings indicate that prior SARS-CoV-2 infection before or shortly after primary vaccination influenced the immune response to mRNA vaccination through immune imprinting, and that repeated exposures to SARS-CoV-2 antigens through vaccination and/or infection may have led to immune damping over time. In contrast, a durable SARS-CoV-2-specific T cell response was observed in the majority of study participants and most likely provided protection against severe infection. These results highlight the complexity of the immune response to SARS-CoV-2 following multiple exposures to viral antigens through infections and/or mRNA vaccinations.

## Figures and Tables

**Table 1:** Anti-RBD antibody titers following primary BNT162b2 vaccination.

		Months [median (IQR) days] from primary BNT162b2 vaccination			
	N. (%)	1.5 [40 (38–42)]	3 [89 (88–91)]	7 [215 (214–220)]	9 [279 (276–280)]
All subjects	258 (100)	258 (100)	253 (98)	252 (98)	236 (91)
		
		Estimated Anti-RBD Antibody Titers (AU/mL), GMT (95% CI) ^a^			
Previously infected subjects ^b^	50 (19)	15,095 * (11,486–19,840)	8259 * (6347–10,746)	2227 * (1707–2905)	1613 * (1218–2136)
Naïve subjects ^c^	208 (81)	8038 (7061–9149)	3023 (2652–3466)	792 (695–903)	597 (522–682)

Abbreviations: Anti-RBD: antibodies against the receptor binding domain of the S1 subunit of the spike protein of SARS-CoV-2; AU/mL: Arbitrary Units per milliliter; GMT, geometric mean titer; 95% CI: 95% confidence interval. ^a^ Estimated GMT of anti-RBD antibody titers with adjustments for age, sex, and SARS-CoV-2 breakthrough infections. ^b^ Subjects with documented SARS-CoV-2 infections that occurred before the first study time point (1.5 months after primary BNT162b2 vaccination). ^c^ Subjects without evidence of prior SARS-CoV-2 infection at the first study time point. * Significantly higher anti-RBD antibody titers at indicated time points (*p* < 0.05).

**Table 2:** Anti-RBD antibody titers following the mRNA-1273 booster.

		Months [median (IQR) days] from mRNA-1273 booster			
	N. (%)	4 [97 (96–97)]	7 [188 (183–189)]	10 [286 (285–289)]	16 [503 (502–505)]
All subjects	130 (100)	130 (100)	112 (86)	126 (97)	83 (64)
		
		Estimated Anti-RBD Antibody Titers (AU/mL), GMT (95% CI) ^a^			
Previously infected subjects ^b^	21 (16)	18,484 (12,668–26,970)	14,874 (9888–22,376)	15,701 (10,759–22,913)	9230 (5808–14,669)
Naïve subjects ^c^	109 (84)	24,360 (20,639–28,751)	22,188 (18,541–26,552)	20,082 (16,947–23,798)	18,783 (15,245–23,141)

Abbreviations: Anti-RBD: antibodies against the receptor binding domain of the S1 subunit of the spike protein of SARS-CoV-2; AU/mL: Arbitrary Units per milliliter; GMT, geometric mean titer; 95% CI: 95% confidence interval. ^a^ Estimated GMT of anti-RBD antibody titers with adjustments for sex, age, and SARS-CoV-2 breakthrough infections. ^b^ Subjects with documented SARS-CoV-2 infections that occurred before the first study time point (1.5 months after primary BNT162b2 vaccination). ^c^ Subjects without evidence of prior SARS-CoV-2 infection at the first study time point.

**Table 3:** SARS-CoV-2 breakthrough infections during follow up.

	Months from Primary BNT162b2 Vaccination				
	1.5	3	7	9	N. Breakthrough Infections
**Previously infected subjects** ^a^	50	50	49	44	
N. breakthrough infections	13 ^c^	0	3	4	20
Infection rate (%)	26.0	0.0	6.1	9.1	
**Naïve subjects ^b^**	208	203	203	192	
N. breakthrough infections	0	1	11	4	16
Infection rate (%)	0.0	0.5	5.4	2.1	
**Total breakthrough infections**					**36**
	**Months from mRNA-1273 Booster**				
	**4**	**7**	**10**	**16**	
**Previously infected subjects** ^a^	21	18	21	14	
N. breakthrough infections	9	6	7	4	26
Infection rate (%)	42.9	33.3	33.3	26.0	
**Naïve subjects ^b^**	109	94	105	69	
N. breakthrough infections	35	22	47	30	134
Infection rate (%)	32.1	23.4	44.8	43.5	
**Total breakthrough infections**					**160**

^a^ Subjects with documented SARS-CoV-2 infection that occurred before the first study time-point (1.5 months following primary vaccination with BNT162b2). ^b^ Subjects without evidence of prior SARS-CoV-2 infection at the first study time point. ^c^ Infections that occurred between the first dose and 1.5 months after the second dose of BNT162b2 primary vaccination, detected by the presence of anti-N antibodies at the first study time point; all were asymptomatic.

**Table 4:** SARS-CoV-2-specific T cell response.

	1st Measurement ^a^	2nd Measurement ^b^
N. tested	107	83
T-cell response		
N. (%) positive	102 (95)	76 (92)
N. (%) negative	5 (5)	7 (8)

^a^ 16 months after the 2nd dose of BNT162b2 (6 months after the mRNA-1273 booster); ^b^ 25 months after the 2nd dose of the BNT162b2 (16 months after the mRNA-1273 booster).

## Data Availability

The data presented in this study are available on request from the corresponding author.
